# Timeliness of Microbiology Test Result Reporting and Association with Outcomes of Adults Hospitalised with Unspecified Pneumonia: A Data Linkage Study

**DOI:** 10.1155/2022/9406499

**Published:** 2022-07-20

**Authors:** Anil Shrestha, Andrew Georgiou, Nasir Wabe

**Affiliations:** ^1^Department of Health Systems and Populations, Faculty of Medicine, Health, and Human Science, Macquarie University, North Ryde, New South Wales, Australia; ^2^Department of Microbiology, Laverty Pathology, North Ryde, New South Wales, Australia; ^3^Centre for Health Systems and Safety Research, Australian Institute of Health and Innovation, Macquarie University, North Ryde, New South Wales, Australia

## Abstract

**Background:**

Pneumonia is one of the leading causes of mortality and morbidity worldwide. Microbiology tests play a critical role in the diagnosis of pneumonia. Our study aimed to determine microbiology result reporting times and evaluate their association with outcomes of adult patients (≥18 years) hospitalised with pneumonia.

**Methods:**

This is a 3-year (2016–2018) retrospective cohort study in six hospitals in New South Wales, Australia. The study data were obtained by linking hospital and laboratory system databases. Result reporting times including time from admission to the first and the last microbiology test results were determined. The outcome measures were hospital length of stay (LOS) and in-hospital mortality. We fit median and logistic regression to evaluate the association of time-to-first microbiological result with hospital LOS and in-hospital mortality, respectively.

**Results:**

A total of 6,298 patients met the inclusion criteria. Of these, 85.3% (*n* = 5,375) ordered at least one microbiology test. The top 5 microbiology tests were blood culture, urine culture, respiratory polymerase chain reaction (PCR), urine antigen, and sputum culture. The median time-to-first microbiology result was 26 hrs while the median time-to-last test result was 144 hrs. The rate of in-hospital mortality was 5.9% (*n* = 371). After adjusting for confounders, every 5 hrs increase in the time-to-first microbiology test was associated with an increase of 3.9 hrs in the median hospital LOS [95% Confidence Interval (CI), 3.5 to 4.3; *P* ≤ 0.001]. There was no association between time-to-first microbiology result and in-hospital mortality (OR 1.01; 95% CI 1.00–1.02; *P*=0.122).

**Conclusion:**

Time-to-first microbiology result reporting was significantly associated with hospital LOS but not with in-hospital mortality. Further research should be conducted to understand if improving result reporting times can reduce the length of hospital stay of patients.

## 1. Introduction

Pneumonia is one of the leading causes of mortality and morbidity worldwide [1]. Globally, in 2015, it was the fourth most common cause of death [2]. In Australia, an estimated 77,000 patients with pneumonia are admitted to hospital each year [3]. The trend of pneumonia deaths in Australia has been increasing [4]. In 2010, the total number of deaths due to pneumonia was 2,373 which was the 15th leading cause of death in the year [4]. In 2014, the total number of deaths due to pneumonia increased to 2,873 which was the 13th most cause of death in the year [4]. In 2019, pneumonia was the 9th most death causing disease with total deaths of 4,124 [4].

Several microbiological laboratory tests can be performed for the diagnosis of pneumonia and detection of etiological agents [5]. These may include culture-based tests such as blood, urine, and sputum cultures, Gram staining, serology tests, and molecular tests using polymerase chain reaction (PCR) [5]. These tests have different laboratory processing times and have different turnaround times (TATs) [6].

Laboratory test TAT is defined as the interval between sample receipt in the laboratory and test report generation [7]. Time to report microbiology test results (e.g., laboratory test TAT, time from admission to test results) can impact patient outcomes in many ways [6]. In one study, delays in laboratory test results in the emergency department (ED) led to a reduction of LOS from 4.1 to 3.2 hours due to a decrease in laboratory TAT outliers percentage from 14.4% to 4.9%, where outliers percentages were defined as the number of tests which had TAT greater than the standard TAT as defined in the study [8]. A longer TAT for microbiological test results may lead to a longer LOS in a hospital, which can lead to patient harm [9]. Previous studies have evaluated the associations between biochemical test TAT with LOS and in-hospital mortality [8, 10]. To the best of our knowledge, there has been no research on the relationships between microbiological test reporting times and patient outcomes such as hospital LOS and in-hospital mortality. The aim of the study is to determine microbiology test ordering patterns, test result reporting times, and their association with hospital LOS and in-hospital mortality among adult patients (aged ≥18 years) admitted to hospitals with unspecified pneumonia.

## 2. Methodology

### 2.1. Study Design and Setting

This retrospective observational (data linkage) study was conducted across six public hospitals in New South Wales (NSW), Australia. The study period was from January 1, 2016, to December 31, 2018. Three of the hospitals (Hospitals A, B, and C) are located within Sydney metropolitan area and the other three hospitals (Hospitals D, E, and F) are located in Illawarra Shoalhaven region. According to the Australian Institute of Health and Welfare's hospital peer groupings, three of the study hospitals are classified as *principal referral*, two hospitals are classified as *public acute group A*, and one hospital is classified as *public acute group B*. Principal referral hospitals are among the largest hospitals in the Australian health system and provide a very broad range of services, including ED, intensive care unit (ICU), and several other specialised units. Public acute group A and group B hospitals are relatively large but do not provide the same range of services as principal referral hospitals [11].

### 2.2. Participants

The patient inclusion criteria were as follows: (1) age ≥18 years and (2) admission with unspecified pneumonia as a principal diagnosis. Unspecified pneumonia was identified using the *International Classification of Disease Version 10 Australian Modification* (ICD-10-AM) J18.9.

### 2.3. Data Sources

The study utilised existing administrative hospital. Comprehensive data on patient demographics, clinical information, and test utilisation were obtained by linking the Laboratory Information System (LIS) and the Admitted Patient Data Collection (APDC). The LIS contains data on laboratory test utilisation (e.g., blood culture orders, dates, and times of test ordering). The APDC contains data on hospital admissions (e.g., diagnosis codes, mode of separation).

### 2.4. Variables

The study outcome measures were *hospital LOS* and *in-hospital mortality*. *Hospital LOS* was defined as the total duration of a patient's stay in hospital, which is the interval between hospital admission and discharge. *In-hospital mortality* is defined as the death of the patient within the hospital during the same episode of admission.

The main independent variables of interest were the microbiological test result reporting times including *time-to-first microbiological result*, *time-to-last microbiology result*, and *laboratory TATs. Time-to-first test result* is defined as the duration from the patient's admission to the hospital to their first microbiological test result becoming available. Similarly, the *time-to-last microbiological test* was defined as the duration from a patient's admission to the hospital to the last microbiological test result becoming available. *Laboratory TAT* for a given test was calculated as the duration between the receipt of a sample in the laboratory and the availability of the test report. We reported laboratory TAT for the top five commonly ordered microbiological tests.

The potential confounders considered in the study included age, gender, Charlson comorbidity index, Diagnosis Related Groups (DRG) complexity, number of tests ordered, source of referral, urgency of admission, repeat microbiological tests ordering, types of microbiological tests ordered, and the hospital of admission. The updated version of the Charlson comorbidity index was calculated based on the ICD-10-AM codes [12].

### 2.5. Statistical Analysis

Descriptive statistics were conducted as appropriate. Microbiology result reporting times (i.e., time-to-first microbiological result, time-to-last microbiology result, and laboratory TATs) were presented using box plots. In the statistical modelling, the *time-to-first microbiological result* was used as a key predictor variable. Time-to-last microbiological test result was not used because most patients either were discharged, had left the hospital, were transferred to another setting, or were deceased before the last test result was available. Similarly, given that most patients can have multiple tests ordered, it would not be practical to find an association between the laboratory TAT of each test with study outcome variables. For interpretation purposes, the time-to-first microbiological result was presented as a 5-hourly interval.

Binary logistic regression was used to determine the association between the time-to-first microbiological result and in-hospital mortality while median regression was used to determine the association between time-to-first microbiological result and hospital LOS. We utilised median regression as opposed to the traditional linear regression because hospital LOS data was highly skewed with a nonnormal distribution. All analysis was adjusted for relevant confounding variables.

### 2.6. Ethical Approval

This study has received ethical approval from the Human Research Ethics Committee of the South Eastern Sydney Local Health District (reference no. HREC/16/POWH/412) and was ratified by Macquarie University.

## 3. Results

### 3.1. Baseline Characteristics

A total of 6,298 patients (51.2% male; median age, 79 years) fulfilled the inclusion criteria.  Table 1 represents the baseline characteristics of patients. Of the 6,298 patients, 85.35% (*n* = 5,375) received at least one microbiological test. The median number of total laboratory tests ordered (including nonmicrobiological tests) was 10 (IQR 7–13) while the median number of microbiological tests ordered was 3 (IQR 1–4) (Table 1).

### 3.2. Microbiology Test Ordering Patterns

**Figure 1:**
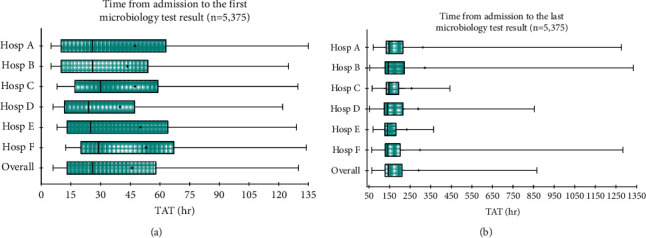
Time from admission to the first (a) and the last (b) microbiology results. (a) Quartile range of time from hospital admission to the first microbiology test result (TAT) with hospitals. Range, upper and lower quartile (box), and mean (+) and median (solid line). (b) Quartile range of time from hospital admission to the last microbiology test result (TAT) with hospitals. Range, upper and lower quartile (box), and mean (+) and median (solid line).

The total number of microbiological tests performed across the six hospitals was 18,608. The top five microbiological tests were blood culture, urine microscopy culture susceptibility (MCS), respiratory PCR, urine antigen, and sputum MCS (Figure 1). These tests accounted for 70.5% (*n* = 13,111) of the total microbiology tests.  Table 2 compares the ordering rates for the top five microbiological tests across the six study hospitals. Of the 6,298 patients, blood culture (*n* = 4012, 63.7%), urine MCS (*n* = 2,786, 44.2%), respiratory PCR (*n* = 2,196, 34.9%), urine antigen (*n* = 2,176, 34.6%), and sputum MCS (*n* = 1,939, 30.8%) tests were ordered across all hospitals. There were some variations in the utilisation of these tests across hospitals (Table 2).

### 3.3. Microbiology Test Result Reporting Times

Of the 5,375 patients who received at least one microbiological test, 86.3% (*n* = 4,641) received the first test results before hospital discharge. However, the proportion of patients for whom the last test results were available before disposition was only 35.5% (*n* = 1,908). The overall median time-to-first microbiological result was 26 hours (IQR, 13–58 hours) (Figure 1(a)). The overall median time-to-last microbiological result was 144 hours (IQR, 128–211 hours) (Figure 1(b)).

Of the five tests, blood culture had the longest laboratory TAT, with a median of 135.8 hours (IQR, 127.9–141 hours) (Supplementary Figure 1A). The test with the shortest laboratory TAT was the urine antigen test (legionella/pneumococcal antigen test), with a median of 3.1 hours (IQR 1.8–7.1 hours) (Supplementary Figure 1D).

### 3.4. Patient Outcomes

The overall median LOS of patients who had at least one microbiological test ordered and were discharged before the first microbiological test result was available was 47 hours (IQR, 20–79 hours). The overall median LOS of patients who had at least one microbiological test and were not discharged from the hospital when the first test result was received was 133 hours (IQR, 84–218 hours) (Table 3). The in-hospital mortality rate was 7.4% among patients who did not receive any microbiology tests. Of patients who received at least one microbiological test, 46 (6.3%) died before receiving the first test result, while 257 (5.5%) died after receiving the first test result (Table 4).

There was an association between the time-to-first microbiology test result and patient outcomes.

This analysis was conducted among patients for whom the first microbiology test results were available before hospital disposition (*n* = 4,641).  Table 5 presents the results of multivariate analysis of factors associated with the study outcomes. The time-to-first microbiology test result was strongly associated with hospital LOS. The multivariate analysis results showed that every 5 hrs increase in the time-to-first microbiology test was associated with an increase of 3.9 hrs in the median hospital LOS (95% CI, 3.5 to 4.3; *P* ≤ 0.001). However, there was no significant association between the time-to-first test result and in-hospital mortality after adjusting for confounding variables (OR 1.01; 95% CI 1.00–1.02; *P*=0.122) (Table 5).

## 4. Discussion

### 4.1. Key Findings

The key finding of this study is that there was a significant association between the time-to-first microbiology result and hospital LOS. For every 5-hour increase in the time-to-first test result, there is an increase in median hospital LOS by 3.9 hours, after adjusting for confounding variables. However, there was no association between the time-to-first microbiology result and in-hospital mortality.

### 4.2. Interpretation and Comparison with Existing Literature

To the best of our knowledge, no prior published studies have evaluated the association between the time-to-first microbiology result and patient outcomes in patients with unspecified pneumonia. The previous studies have been conducted in the context of broader patient populations in EDs using laboratory TAT as the key indicator [9, 13]. Therefore, we cannot make direct comparisons with other studies. A retrospective study of four hospitals in Sydney, Australia, from 2008 to 2011, by Li et al. found that, for every 60-minute increase in laboratory TAT, there was an increase in ED LOS of 35 minutes [13]. A similar study by Kaushik et al. in the United States also found a significant association between ED LOS and TAT [9]. They found that, for every 1-minute decrease in laboratory TAT, there was a 0.50-minute decrease in ED LOS [9].

Although the studies by Li et al. and Kaushik et al. support our finding of a significant association between the timing of laboratory test results and LOS, the differences in the increase in the time may be because our study focused on specific disease (i.e., unspecified pneumonia) rather than broader patient population [9, 13]. The differences could also be due to differences in the indicator of reporting time studied (we used the time-to-first microbiological instead of the laboratory TATs). Also, the studies of Li et al. and Kaushik et al. examined ED patients, whereas we examined general patients admitted to the hospital [9, 13].

Our study also found that, for every five microbiological tests ordered, the hospital LOS of pneumonia patients increased by 48.3 hours. A study by Li et al. also found a significant association between the number of additional tests ordered and ED LOS [13]. They found that, for every five additional tests, the LOS increased by 10 minutes [13]. However, there were much higher differences in the LOS vs additional test relationships reported in our study than those of Li et al. [13]. This may be because, in our study, we used microbiological tests that take longer, whereas those considered by Li et al. involved laboratory tests such as clinical chemistry, molecular genetics, immunology, haematology, anatomical pathology, blood bank, and endocrinology, which have relatively short laboratory TATs [13]. Furthermore, Li et al. investigated ED patients, whereas our study only considered patients admitted to hospitals with pneumonia [13]. Patients in an ED usually have shorter LOSs than hospital-admitted patients. Therefore, differences in study setting and laboratory tests might be responsible for the greater differences in the LOS vs additional test relationships reported by our study compared to that of Li et al. [13].

Our study found no significant association between the time-to-first microbiological test and in-hospital mortality. This indicates that other factors such as patient age or ICU admission, instead of the time-to-first microbiology results, are important determinants of in-hospital mortality.

### 4.3. Implications for Practice

This study has provided evidence that an increase in the time from admission to the first test result is associated with an increase in hospital LOS. This time increase can be in any stage, that is, time taken for a physician to examine the patients, order a microbiological test, and collect a sample (preanalytical stage), and for the laboratory to process the sample (processing stage) and disseminate the results (postanalytical stage). The findings of this study imply that if the time frame from physician check-up to laboratory result dissemination of the first microbiological test result, at any stage, can be minimised, then the LOS of patients in hospital can be decreased. The findings of this study are critical to inform any future intervention studies aiming to reduce hospital LOS by optimising the timing of care in hospitals.

### 4.4. Strengths and Limitations of the Study

To the best of our knowledge, this is the first study to investigate the association between the time-to-first microbiological result and patient outcomes in hospitals. The key strengths of the study are its large sample size and utilisation of data from several hospitals.

The main limitation of this study is that as this is an observational study, unmeasured factors can be a potential confounder of its outcomes. For example, this study did not have data on the pneumonia severity index (PSI) which may have impact on the study outcomes. One important limitation is that we did not assess the effects of microbiology test results (positive or negative) as we did not have access to such data. Another limitation of the study is that the degree of severity of symptoms has not been included in the study because we did not have data on severity of symptoms.

## 5. Conclusion

This study found a significant association between the time-to-first microbiological test result and hospital LOS in adult patients admitted with unspecified pneumonia. Further study should be conducted to understand if improving result reporting times can reduce the length of hospital stay of patients. However, this study did not find statistically significant association between the time-to-first microbiology test result and in-hospital mortality.

## Figures and Tables

**Table 1:** Comparison of the characteristics of patients by microbiology test order status 2016–2018.

	Total	Microbiology test ordered	
		No	Yes
Gender	6,298 (100)	923	5,375
Male	3,222 (51.2	423 (13.1)	2799 (86.9)
Female	3,076 (48.8)	500 (16.3)	2576 (83.8)

Age in years, median (IQR)	79 (68–86)	80 (68–87)	79 (67–9)

Age group in years			
≤65	1,412 (22.4)	193 (13.7)	1219 (86.3)
66–75	1,141 (18.1)	144 (12.6)	997 (87.4)
76–85	1,951 (31)	290 (14.9)	1,661 (85.1)
≥86	1,794 (28.5)	296 (16.5)	1,498 (83.5)

Source of referral			
ED	5,931 (94.2)	810 (13.7)	5,121 (86.3)
Other (e.g., medical practitioner)	367 (5.8)	113 (30.8)	254 (69.2)

Urgency on admission			
Urgent	6,074 (96.4)	846 (13.9)	5,228 (86.1)
Nonurgent	224 (3.6)	77 (34.4)	147 (65.6)

Number of laboratory tests ordered, median (IQR)	10 (7–13)	4 (2–6)	10 (8–13)

Number of microbiology tests ordered, median (IQR)	3 (1–4)	—	3 (1–4)

AR-DRG complexity			
Minor/intermediate	2,064 (32.8)	413 (20)	1,651 (80)
Major	4,234 (67.2)	510 (12.1)	3,724 (88)

Procedure conducted			
No	1,128 (17.9)	310 (27.5)	818 (72.5)
Yes	5,170 (82.1)	613 (11.9)	4,557 (88.1)

Charlson comorbidity index, median (IQR)	7 (4–11)	5 (3–9)	7 (4–11)

Charlson comorbidity index			
0	1,572 (25)	265 (16.9)	1,307 (83.1)
1	1,428 (22.7)	217 (15.2)	1,211 (84.8)
2	1,172 (18.6)	159 (13.6)	1,013 (86.4)
>2	2,126 (33.8)	282 (13.3)	1,844 (86.7)

Year of admission			
2016	1,972 (31.3)	291 (14.8)	1,681 (85.2)
2017	2,121 (33.7)	311 (14.7)	1,810 (85.3)
2018	2,205 (35.0)	321 (14.6)	1,884 (85.4)

Hospital			
STG	1,901 (30.2)	285 (15)	1,616 (85.0)
POW	1,221 (19.4)	238 (19.5)	983 (80.5)
TSH	1,219 (19.4)	157 (12.9)	1,062 (87.1)
WOL	1,083 (17.2)	133 (12.3)	950 (87.7)
SHH	471 (7.5)	70 (14.9)	401 (85.1)
SHV	403 (6.4)	40 (9.9)	363 (90.1)

**Table 2:** Comparison of the top five microbiology test ordering rates across hospitals.

Hospital	Total *n*	Blood culture	Urine MCS	Respiratory PCR	Urine antigen^*∗*^	Sputum MCS
A	1,901	1261 (66.3)	702 (36.9)	667 (35.1)	690 (36.3)	587 (30.9)
B	1,221	703 (57.6)	537 (44.0)	442 (36.2)	348 (28.5)	357 (29.2)
C	1,219	835 (68.5)	569 (46.7)	367 (30.1)	402 (33.0)	328 (26.9)
D	1,083	653 (60.3)	570 (52.6)	434 (40.1)	437 (40.4)	390 (36.0)
E	471	298 (63.3)	230 (48.8)	155 (32.9)	152 (32.3)	141 (29.9)
F	403	262 (65.0)	178 (44.2)	133 (33.0)	147 (36.5)	136 (33.7)
Total	6,298	4012 (63.7)	2,786 (44.2)	2,198 (34.9)	2,176 (34.6)	1,939 (30.8)

^*∗*^Legionella/pneumococcal; MCS, microscopy culture and sensitivity; PCR, polymerase chain reaction.

**Table 3:** Patient outcomes (hospital length of stay). A: patient disposition occurred before the first test results were available. B: patient disposition occurred after the first test results were available.

Hospitals	Hospital length of stay (hr), median (IQR)		
	Microbiology test ordered		
	No (*n *= 923)	Yes	
		A (*n *= 734)	B (*n *= 4,641)
A	65 (20–132)	41 (20–76)	124 (77–213)
B	60 (11–112)	23 (12–57)	123 (76–197)
C	71 (27–126)	56 (23–77)	141 (84–219)
D	95 (72–148)	72 (42–100)	145 (97–246)
E	120 (74–170)	65 (36–99)	124 (88–197)
F	97 (59–150)	73 (48–105)	139 (91–238)
Overall	75 (27–136)	47 (20–79)	133 (84–218)

**Table 4:** Patient outcomes (in-hospital mortality). A: patient disposition occurred before the first test results were available. B: patient disposition occurred after the first test results were available.

Hospitals	In-hospital mortality, n (%)		
	Microbiology test ordered		
	No (*n* = 923)	Yes	
		A (*n* = 734)	B (*n* = 4,641)
A	19 (6.7)	11 (4.0)	79 (5.9)
B	12 (5.0)	10 (6.2)	42 (5.1)
C	17 (10.8)	11 (8.0)	53 (5.7)
D	11 (8.3)	8 (12.1)	53 (6.0)
E	4 (5.7)	3 (7.3)	10 (2.8)
F	5 (12.5)	3 (5.6)	20 (6.5)
Total	68 (7.4)	46 (6.3)	257 (5.5)

**Table 5:** Factors associated with hospital LOS and in-hospital mortality (multivariate analysis).

	Change in median LOS (hr)		In-hospital mortality	
	Coefficient (95% CI)	*P*-value	OR (95% CI)	*P*-value
Time-to-first test results (for every 5 hrs increase)	3.9 (3.5–4.3)	≤0.001	1.01 (1–1.02)	0.122

Age (for every 10-year increase)	6.1 (3.8–8.3)	≤0.001	1.68 (1.46–1.94)	≤0.001

Source of referral				
ED vs other	−24.1 (−39.5– (−8.7))	0.002	0.57 (0.33–0.98)	0.043

ICU/HDU admission				
Yes vs no	26 (13.3–38.7)	≤0.001	4.16 (2.83–6.12)	≤0.001

Procedure conducted				
Yes vs no	29 (18.1–40.1)	≤0.001	0.68 (0.39–1.17)	0.166

Charlson comorbidity index				
0	Reference		Reference	
1	−3.7 (−13.4–5.9)	0.445	1.21 (0.67–2.20)	0.529
2	2.1 (−8.1–12.3)	0.683	1.76 (1.01–3.06)	0.045
>2	21.9 (12.5–31.3)	≤0.001	3.61 (2.20–5.92)	≤0.001

DRG complexity				
Major vs minor/intermediate	22.2 (14.1–30.4)	≤0.001	1.60 (1.05–2.45)	0.029

No. of tests ordered (for every 5 more tests)	48.3 (43.8–52.8)	≤0.001	1.25 (1.06–1.46)	0.007

Repeat microbiology test requested				
Yes vs no	20.8 (13.6–28)	≤0.001	1.08 (0.79–1.46)	0.637

Blood culture ordered				
Yes vs no	−15.4 (−23– (−7.8))	≤0.001	1.54 (1.08–2.19)	0.017

Respiratory PCR ordered			—	—
Yes vs no	4.2 (2.7–11.1)	0.235	—	—

Urine MCS ordered				
Yes vs no	2.7 (−4.3–9.8)	0.445	1.36 (0.99–1.85)	0.055

Urine antigen test ordered^*∗*^				
Yes vs no	−5.8 (−13.1–1.5)	0.120	0.79 (0.58–1.07)	0.129

Hospital				
A	*Reference*		*Reference*	
B	−5.2 (−14.8–4.4)	0.290	0.90 (0.60–1.35)	0.607
C	1.2 (−8.1–10.4)	0.806	0.85 (0.58–1.24)	0.393
D	−9.2 (−18.9–0.6)	0.066	0.85 (0.58–1.26)	0.417
E	−28.3 (−41.4– (−15.2))	≤0.001	0.38 (0.19–0.76)	0.006
F	−28.9 (−42.8– (−15))	≤0.001	0.65 (0.38–1.12)	0.118

LOS: length of stay. ^*∗*^legionella/pneumococcal.

## Data Availability

The study utilised existing administrative hospital. Comprehensive data on patient demographics, clinical information, and test utilisation were obtained by linking the Laboratory Information System (LIS) and the Admitted Patient Data Collection (APDC). The LIS contains data on laboratory test utilisation (e.g., blood culture orders, dates, and times of test ordering). The APDC contains data on hospital admissions (e.g., diagnosis codes, mode of separation).

## Additional Points

(i) What is already known about the topic? Laboratory turnaround time is the time interval between the sample receipt in laboratory and dissemination of that laboratory test result. From previous studies, it was found that delay in laboratory turnaround time increases the length of stay of patients in hospital [8]. (ii) What does the article add? This study has found association between time-to-first microbiological test result with the length of stay of pneumonia patients in hospital. The study found that, for every 5-hour increase in the time-to- first microbiological test result, there is an increase in median hospital length of stay by 3.9 hours.

## Supplementary Materials

Supplementary Materials Supplementary Figure 1 (A–E) provides information on turnaround times of top five microbiology tests ordered in this study. The supplementary figures provide detailed information on the number of tests ordered in different hospitals and their turnaround time.
