# Supplementary material for: Timeliness of Microbiology Test Result Reporting and Association with Outcomes of Adults Hospitalised with Unspecified Pneumonia: A Data Linkage Study
Source: Int J Clin Pract. 2022 Jul 20;2022:9406499. doi: 10.1155/2022/9406499 (PMC9328961; doi:10.1155/2022/9406499)
Supplement: Supplementary Materials — Supplementary Figure 1 (A–E) provides information on turnaround times of top five microbiology tests ordered in this study. The supplementary figures provide detailed information on the number of tests ordered in different hospitals and their turnaround time. [file 9406499.f1.zip › supplementary figures.docx]

**List of Supplementary Figures.**

**Supplementary Figure 1: The turnaround times (A-E) of the top five microbiology tests.**

**A B**

**C**

**D E**

Figure 2A-E: Quartile range of turnaround time of microbiology tests result (TAT) with hospitals. Range, upper and lower quartile (box), mean (+) and median (solid line).
