# Supplementary figures and images for: Timeliness of Microbiology Test Result Reporting and Association with Outcomes of Adults Hospitalised with Unspecified Pneumonia: A Data Linkage Study
Source: Int J Clin Pract. 2022 Jul 20;2022:9406499. doi: 10.1155/2022/9406499 (PMC9328961; doi:10.1155/2022/9406499)

# Blood culture (n=4,012)

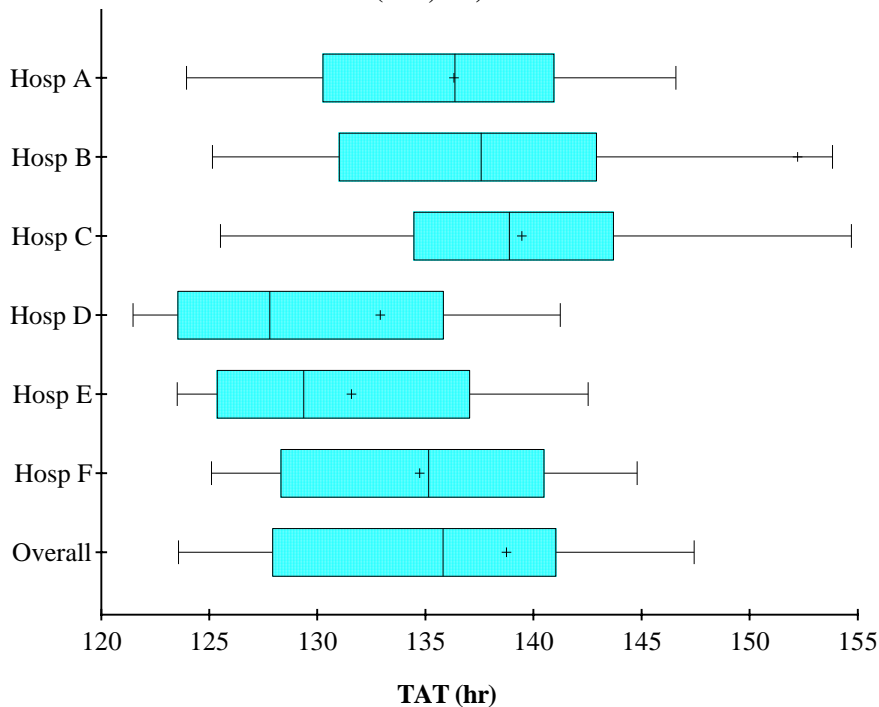

Supplement: Supplementary Materials — Supplementary Figure 1 (A–E) provides information on turnaround times of top five microbiology tests ordered in this study. The supplementary figures provide detailed information on the number of tests ordered in different hospitals and their turnaround time. [file 9406499.f1.zip › Figure S1A (1).pdf]

# Urine MCS (n=2,786)

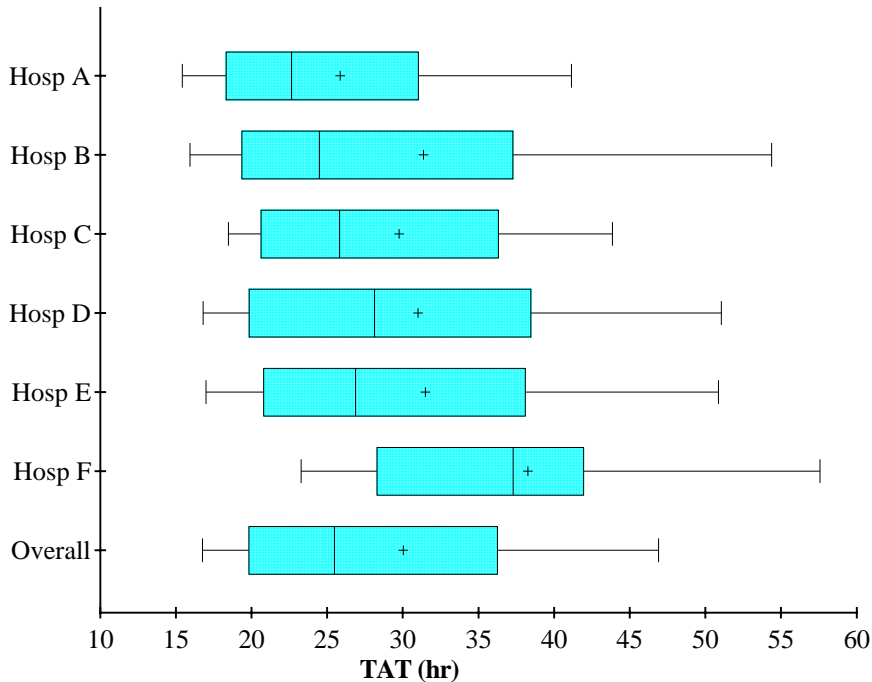

Supplement: Supplementary Materials — Supplementary Figure 1 (A–E) provides information on turnaround times of top five microbiology tests ordered in this study. The supplementary figures provide detailed information on the number of tests ordered in different hospitals and their turnaround time. [file 9406499.f1.zip › Figure S1B (1).pdf]

## Respiratory PCR (n=2,198)

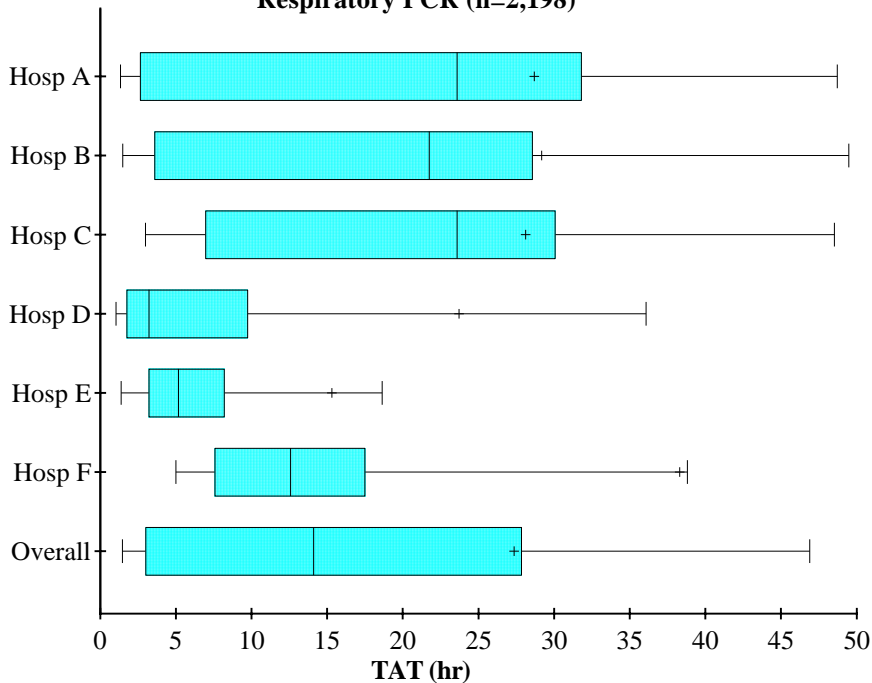

Supplement: Supplementary Materials — Supplementary Figure 1 (A–E) provides information on turnaround times of top five microbiology tests ordered in this study. The supplementary figures provide detailed information on the number of tests ordered in different hospitals and their turnaround time. [file 9406499.f1.zip › Figure S1C (1).pdf]

# Urine antigen (n=2,176)

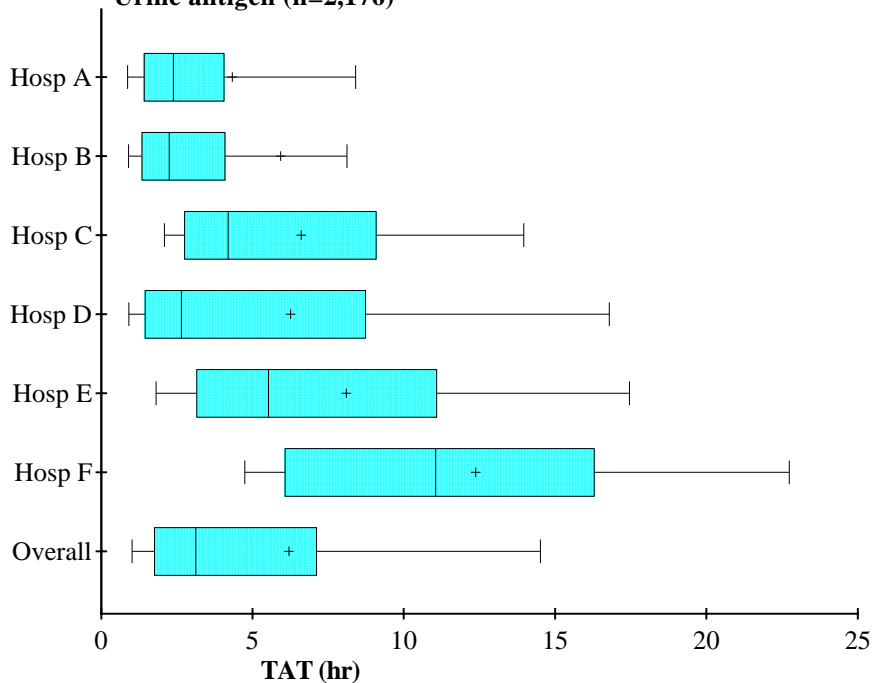

Supplement: Supplementary Materials — Supplementary Figure 1 (A–E) provides information on turnaround times of top five microbiology tests ordered in this study. The supplementary figures provide detailed information on the number of tests ordered in different hospitals and their turnaround time. [file 9406499.f1.zip › Figure S1D (1).pdf]

# Sputum MCS (n=1,939)

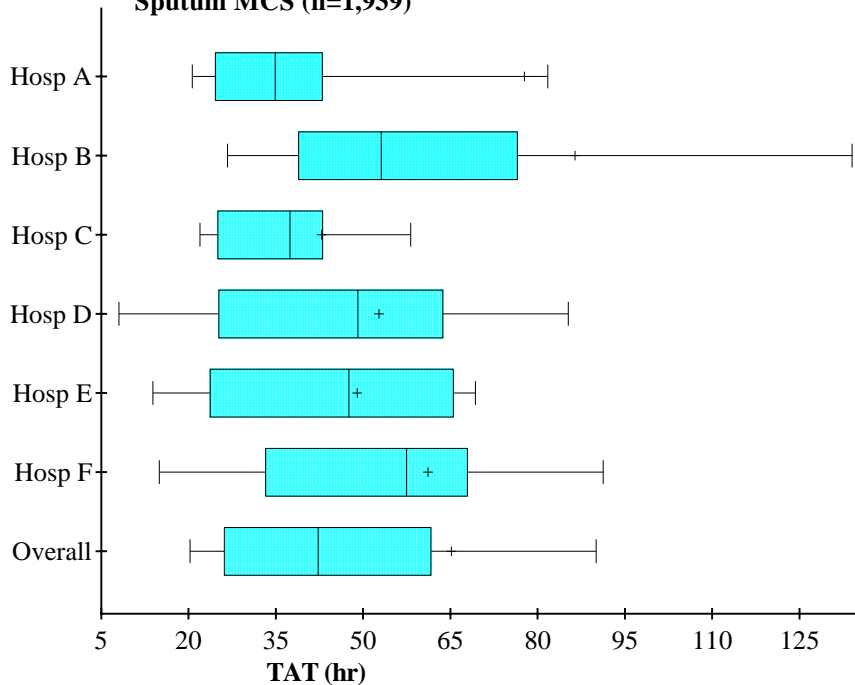

Supplement: Supplementary Materials — Supplementary Figure 1 (A–E) provides information on turnaround times of top five microbiology tests ordered in this study. The supplementary figures provide detailed information on the number of tests ordered in different hospitals and their turnaround time. [file 9406499.f1.zip › Figure S1E (1).pdf]
